# Droplet-Confined Electroplating for Nanoscale Additive Manufacturing: Current Control of the Initial Stages of Growth of Copper Nanowires

**DOI:** 10.1021/acselectrochem.4c00085

**Published:** 2024-11-04

**Authors:** Mirco Nydegger, Ralph Spolenak

**Affiliations:** Laboratory for Nanometallurgy, Department of Materials, ETH Zürich, Vladimir-Prelog-Weg 1-5/10, Zürich 8093, Switzerland

**Keywords:** microscale, additive manufacturing, metal nanostructures, 3D nanofabrication, electrohydrodynamic ejection, EHD

## Abstract

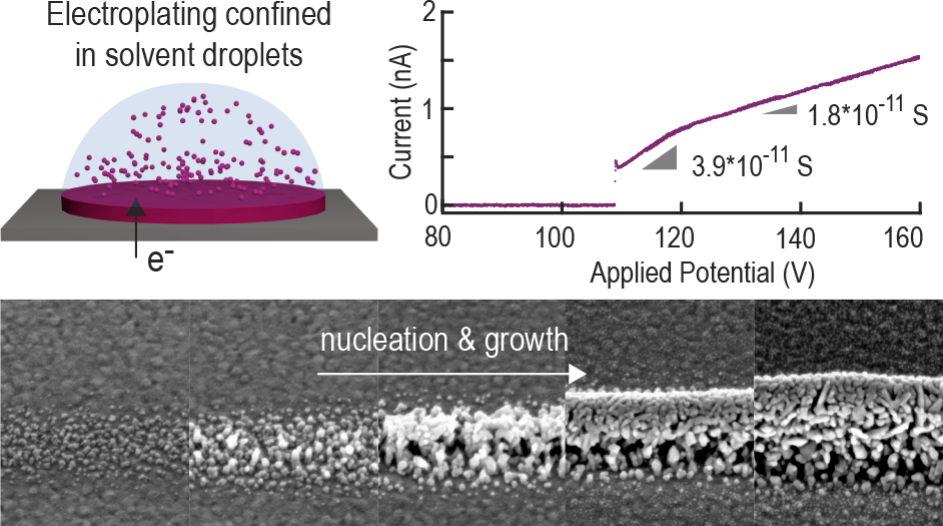

Droplet-confined electrodeposition enables a precise deposition of three-dimensional, nanoscopic, and high purity metal structures. It aspires to fabricate intricate microelectronic devices, metamaterials, plasmonic structures, and functionalized surfaces. Yet, a major handicap of droplet-confined electrodeposition is the current lack of control over the process, which is owed to its dynamic nature and the nanoscopic size of the involved droplets. The deposition current offers itself as an obvious and real-time window into the deposition and needs to be analyzed operando. Nucleation and growth dynamics are evaluated systematically. Our results indicate different deposition regimes and link the current to both morphology and volume of deposited copper. This allows for optimized electroplating strategies and calibration of the slicing algorithms necessary for a controlled deposition of 3D structures with different solvents. The potential of selecting appropriate solvents further readies this novel technique for the reliable deposition of functional structures with submicron resolution.

## Introduction

Intricate, nanostructured metals have exciting applications in integrated circuits and MEMS devices,^7,8^ nanorobots,^9^ and plasmonic structures.^10^ Small-length-scale additive manufacturing (AM) aspires to provide access to such 3D structures at the nanoscale without the geometrical constraints of two-dimensional lithography. Small-length-scale AM techniques based on localized electrodeposition^11,12^ are especially well-suited for the deposition of device-grade metals, due to a high purity and a dense microstructure^13,14^ of plated metals. Such plated metals often exhibit excellent mechanical and electrical properties.^14^ Therefore, small-length-scale electrochemical AM has drawn significant attention from both fundamental and industrial research. There are different approaches to small-length-scale electrochemical AM techniques that all rely on a localization of one of the fundamental steps of electroplating: a localized supply of electrons and cations or a spatial confinement of the electrolyte.

### Localized Supply of Electrons

The localized supply of electrons for reduction can be achieved by using focused electron beams (such as in electron microscope)^15^ or by utilizing sharp electrodes (such as sharp scanning tunneling microscope tips).^16^ Here, the short mean free path of electrons in solvents will spatially constrict the deposition.^17^ Despite the potential for layer-by-layer deposition, the fabrication of arbitrarily shaped, 3D structures with submicron resolution have not yet been demonstrated to our knowledge.

### Localized Supply of Cations

Cations can be provided locally by immersing hollow AFM tips (referred to as FluidFM)^18^ or quartz nozzles in a supporting electrolyte, in a setup similar to scanning ion conductance microscopy (SICM).^19^ This allows to produce high-fidelity structures from a variety of metals.^20^

### Confinement of the Electrolyte

A more common approach is to utilize a mask so that only parts of the conductive substrate are exposed to the electrolyte. This will limit the deposition into the exposed area and leave an out-of-plane structure when the mask is removed. This approach is often used to synthesize nanowires,^9^ but also more intricate structures.^11,21^ Electroplating confined by a meniscus between the substrate and a nozzle offers a flexible mask and therefore a higher flexibility for intricate designs. This technique further allows for the fabrication of high resolution structures (<40 nm).^22^

**Figure 1:**
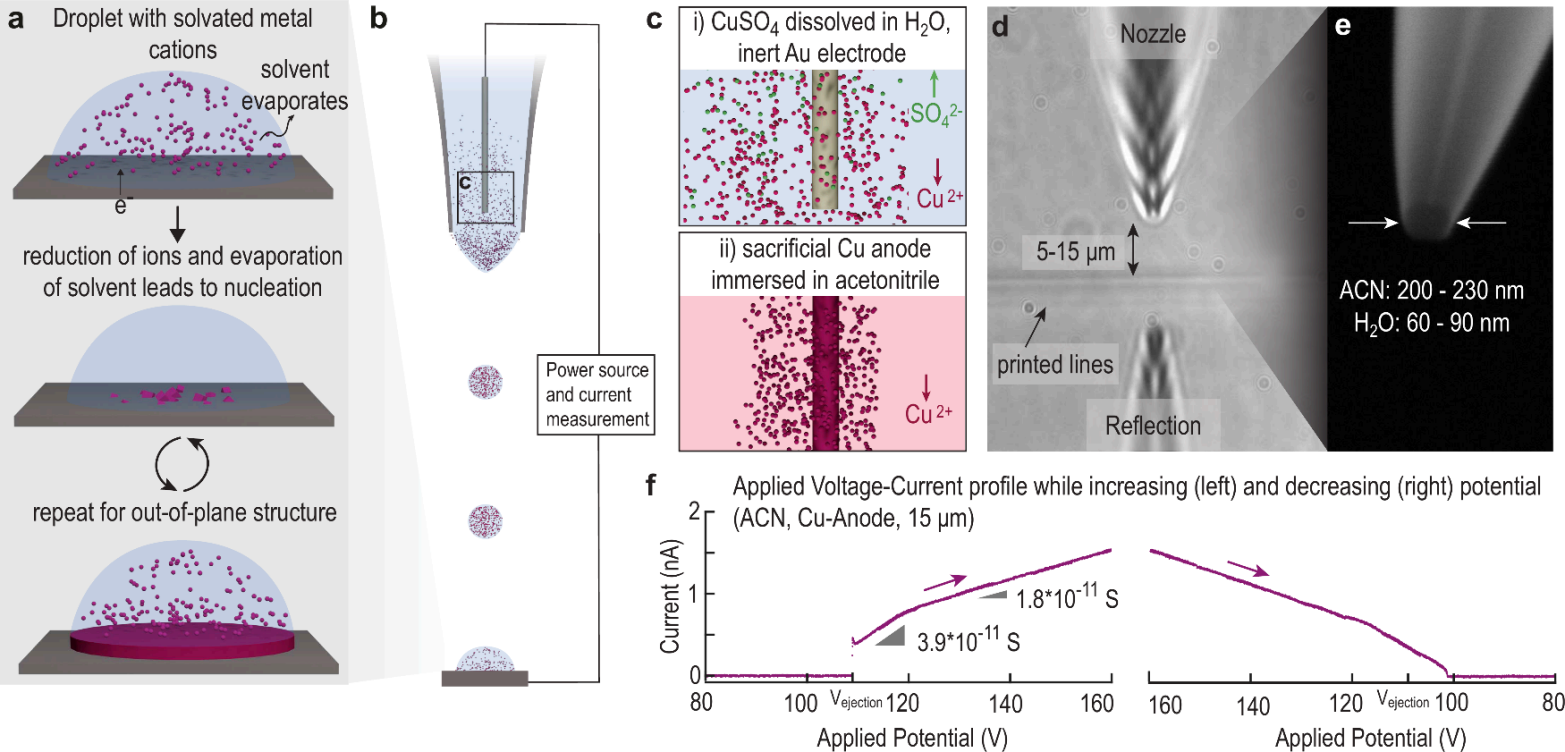
Droplet-confined electroplating utilizing electrohydrodynamic redox 3D printing. (a) We spatially confine electroplating into nanoscopic solvent droplets. It is important to note that these droplets contain only metal-cations and solvent molecules^1,2^. On the substrate, the metal ions are reduced while the solvent evaporates. A repetition of this cycle leads to the formation of an out-of-plane metal nanostructure. (b) The solvent droplets with solvated metal ions can be generated and deposited using electrohydrodynamic (EHD) ejection, which has been described in detail previously.^1,3,4^ Here, a solvent-filled quartz capillary is used as a nozzle, and gold-coated wafers are used as substrates. (c) For the present study, we utilize and compare two different ion sources and solvents that have been successfully used in previous work: dissolved metal salts in low concentrations (1 mM CuSO_4_ in H_2_O)^5^ and sacrificial metal anodes in acetonitrile (ACN).^1,6^ (d) Camera image showing the printing process: the nozzle (top) is in the proximity (5–15 μm) of the substrate. At the bottom, the reflection of the nozzle is visible, while printed structures appear as diffuse lines. (e) Scanning electron micrographs verify the orifice of the nozzle to be between 200 to 230 nm for ACN and 60 to 90 nm for dilute metal salt solutions in H_2_O. (f) Voltammetry of a deposition with ACN as the solvent and a copper anode (scan speed 50 mV/s). We can distinguish different regimes in the *I*–*V* (current–potential) curve with increasing potential (left graph). At *V* < *V*_ejection_, no ejection is observed and therefore a current of zero is measured. At *V* = *V*_ejection_, ejection of ion-loaded droplets starts. The start is not a gradual increase in the current but rather a sudden change to around 0.5 nA. Above a current of around 0.8–1 nA, the slope of the *I*–*V* curve changes: a decrease in the conductance is observed (from 3.9 × 10^–11^ S to 1.8 × 10^–11^ S). When the potential is decreased (right graph), a similar curve is observed with a change in the slope at the same current. However, the curve is continuous until a current of 0 is measured. Therefore, low deposition currents (*I* < 0.5 nA) can be accessed by starting the deposition at a high potential and then reducing the potential again.

A fundamental limitation shared by all electrochemical additive manufacturing (ECAM) techniques is the low deposition speed compared to other small-length-scale AM techniques that rely on transfer of presynthesized materials.^13^ To increase the deposition speed of ECAM techniques, a forced mass-transfer by ejection of ion-loaded solvent droplets in an electric field can be used.^23^ The forced mass-transfer is then followed by a spatially confined electroplating in droplets.^1^ This technique, known as electrohydrodynamic redox 3D printing (EHD-RP), allows for a fabrication of complex structures with speed of around 100 voxels/s, the voxel size being around 60 nm.^6,23^ The voxel size directly depends on the size of the solvent droplet, which is generated from a quartz capillary (Figure 1a–e). Therefore, EHD-RP combines a high deposition speed typical for material-transfer AM techniques with the dense microstructure of electroplated metals. Combining this technique with an electron microscope allows to electroplate metals directly onto insulating substrates.^24^

It is important to note that droplet confinement is conceptually not merely an extension of standard bulk electrochemical processes. In particular, the transient nature of the droplet as well as the absence of a liquid connection between the electrodes are unique features of EHD-RP. This allows, for example, direct deposition of alloys with a controlled chemical composition^5^ without the need for additives. Furthermore, the deposited metal can be switched in a voxel-by-voxel fashion on-the-fly^1^ to create chemically architected materials. Yet, several fundamental questions still need to be addressed to fully leverage this novel technique routinely in nanofabrication. Specifically, to prepare this technique for the fabrication of complex structures, a direct feedback mechanism for the printed volume has to be established. This is fundamental to optimize the discretization of a planned design into individual voxels to be printed (*slicing*) and is therefore crucial for the reliable fabrication of intricate nanostructures.^10^ Further, the nucleation and current-microstructure relationships in this novel regime of electroplating have only partially been described,^6^ despite their importance for the fabrication of metals with dense microstructures. Finally, an accurate current control promises to optimize the resolution of droplet-confined electroplating.

In this work, we first investigate the deposition current by mapping the relationship between applied potential and measured current in EHD-RP for different solvent systems and different distances between the nozzle and substrate. We then investigate the nucleation in droplet-confined electroplating at different currents. Lastly, we establish a relationship between the deposition current and the deposited voxels. We limit the number of voxels that are investigated to 4 (with 50 ms deposition time each) to limit the influence of other effects such as field focusing.^10^

## Results

### Voltammogram of Electrohydrodynamic Redox Printing

**Figure 2:**
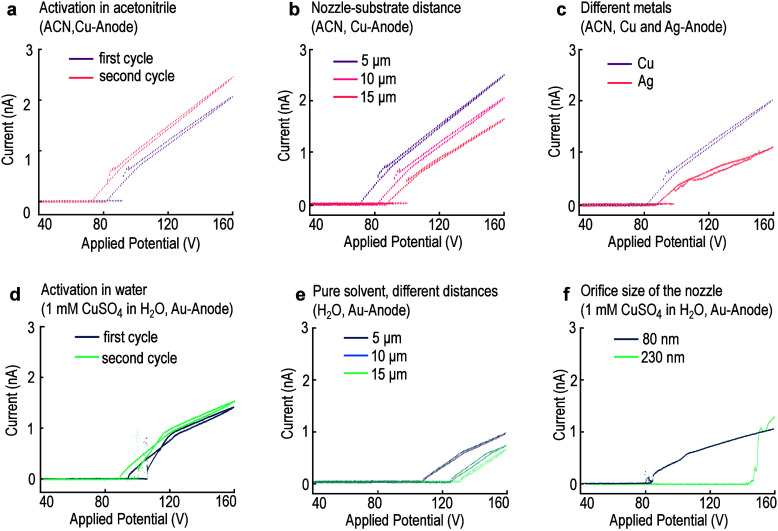
Voltammetry measurements of EHD-RP. (a) Two cycles from 0 to 160 V and back with the same nozzle and electrode do not show the same current values. In the second cycle, the curve is shifted to higher currents for the same potential, indicating an activation of the surface of the electrode. Note that the current at which the slope changes stays constant (scan speed, 500 mV/s). (b) The nozzle-to-substrate distance has a strong influence on the current. A smaller distance yields a higher current for the same potential and lowers the necessary potential to start the ejection. Furthermore, the current at which the resistance increases is higher at smaller distances. (c) The element that is used as a sacrifical anode also has a large influence on the observed current. It is to know that copper oxidizes more readily compared to a silver anode, which leads to a higher current for the same potential. (d) The current-curve of copper sulfate dissolved in water exhibits a similar change in the slope around a current of 0.8–1 nA. The onset of the ejection is unstable with currents observed varying between 0 and 1 nA. A second cycle shifts the curve toward higher current similarly as observed for ACN. (The scan speed is 50 mV/s to illustrate the start of the ejection). (e) Ejection of copper-free water is possible but requires high potentials to start the ejection (presumably of hydroxonium-loaded droplets, that is reduced to hydrogen on the cathode) unless the nozzle is very close to the substrate. Without copper ions present only low currents are achieved, and no unstable current was observed upon start of the ejection (i.e. fluctuating current). Increasing and decreasing the potential yields parallel current-curves. (f) The size of the orifice has a large influence on the ejection when using water-based solvents: small orifices (60–90 nm) enable an ejection starting from a similar potential (ca. 80 V) as in ACN. In contrast, 230 nm nozzles show a very different current–potential behavior with a steep increase in the current after ejection start. This steep increase in current will render the deposition difficult to control; therefore, narrower orifices are used to deposit aqueous electrolytes.

Deposition in EHD-RP is assumed to be confined into transient solvent-droplets without a liquid connection between the working and counter electrode. This unique working principle is reflected by a distinctive shape of the voltammograms (plots of applied potential versus the measured current) of EHD-RP (Figure 1f and Figure 2). The voltammograms are measured by applying a constant potential between the immersed electrode (which acts as the anode in the setup) and the substrate (the cathode) while measuring the current for 1 s. In the present setup and with the applied potentials, electrons flow from the electrode immersed in the solvent in the nozzle to the substrate. Similarly, an equal amount of positive charges are ejected from the nozzle toward the substrate. For the present study, for readability, we notate the current as positive when electrons and cations flow from the nozzle to the substrate. The applied potential is cycled between 0 and 160 V with a Δ*V* of 0.5 V (Figure 1b, resulting in a scan speed of 500 mV/s, unless stated otherwise). For these experiments, the printed geometry consisted of parallel lines while maintaining a substrate–nozzle distance of 10 μm (or between 5–15 μm when investigating the influence of the distance). Note that the measured distance between nozzle and substrate is determined optically and therefore fundamentally limited to a precision of 3–10% for distances between 5 and 15 μm due to the principal resolution limit of the lens (λ = 530 nm and NA = 0.5, hence *d* = 530 nm). To verify that the observed current–potential relationship is not an artifact of the used amperemeter or setup, the anode was replaced with a 100 GOhm resistor (selected to match the current at 100 V) and connected to the substrate. With the wire replaced by a resistor, the current did not show such change in the slope (Supporting Information (SI) Figure S1a). Additionally, no current was detected when no solvent was added to the nozzle. We therefore conclude that the observed *I*–*V* curves are not due to artifacts in the setup.

The *I*–*V* curve in Figure 1f shows three distinct regimes characterized by different slopes of the curve. When a potential below the minimal ejection potential (*V* < *V*_ejection_) is applied, no current above noise level (±2 × 10^–11^ A) is measured. Here, the applied potential is not strong enough to start the hydrodynamic ejection of ion-loaded droplets. Note that in contrast to standard EHD printing no pressure is applied to the liquid in the nozzle.^3^ The absence of a detectable current above noise-level includes the region around 0 (0 ± 4 V) where a current from the dissolution of the Cu electrode could be expected but is not observed (SI Figure S1b). The only current that is detectable for *V* < *V*_ejection_ is an induced current from changing the potential, since the experimental setup exhibits significant capacitive elements. The current induced by the capacitive element decays quickly if Δ*V* is small (Δ*V* ≤ 1 V/*s*). When the potential is raised to above the minimal ejection potential *V*_ejection_, a sudden increase in current is observed. Usually, a potential of 75–90 V is necessary to start ejection. However, this minimal potential depends on further parameters, such as the distance between the nozzle and substrate, ion concentration, and diameter of the nozzle (Figure 2). For 5 μm distance and acetonitrile (ACN) as the solvent, a potential of *V*_ejection_ = 82 V is necessary, for 10 μm *V*_ejection_ = 92 V, and for 15 μm *V*_ejection_ = 100 V. Upon start of the ejection, the current rises instantaneously to ∼0.5 nA for ACN. For water, the current fluctuates in the beginning between 0 and 1 nA (Figure 2c), indicating an unstable ejection mode but then stabilizes at around 0.2 nA. A further increase of the potential leads to a linear increase of the current in both solvents (ACN: 3.9 × 10^–11^ S, 1 mM CuSO_4_: 5 × 10^–11^ S). Above a current of around 0.8–1 nA, the differential conductance (local derivative of *I*/*V*) decreases for both solvents (ACN: 1.8 × 10^–11^ S, 1 mM CuSO_4_: 1.4 × 10^–11^ S). A change in the conductance is observed at the same current when the potential is reduced (Δ*V* < 0, Figure 1f). The main difference with Δ*V* < 0 is that the ejection is continuous until the current is reduced to effectively 0. Therefore, deposition at low currents (*I* < 0.3 nA for water and *I* < 0.5–0.8 nA in ACN) can be achieved by first setting the potential so that a current of <0.5 nA is measured and then lowering the potential again. Droplets can also be ejected with negative potentials as shown in SI Figure S1c, but the ejection is not stable as indicated by the current curve. So far, no structures have been printed using negative applied potentials.

We observed that the current is dependent on a variety of parameters. During the second cycle between 0 and 160 V, a higher current compared to the first cycle is measured for both ACN and water as the solvent (Figure 2a,c). A third and fourth cycle each led to a further increase (SI Figure S1c). An increase in the nozzle-to-substrate distance, however, leads to a decrease in the current and necessitates a higher *V*_ejection_ to start the ejection. Water without an added electrolyte can be ejected with the same three different regimes of the conductance (except for the highest nozzle–substrate distance, where the current stays too low) but requires high potentials to start the ejection. Consequently, an increased concentration of metal ions in the solvent lowers the potential needed to initiate ejection. The orifice size of the nozzle also has a pronounced influence on the ejection behavior. While with a small aperture the above described behavior is observed, a large nozzle orifice necessitates a large potential to start the ejection and the current increases steeply. Large orifices render the control over the current difficult due to this steep increase of the current.

Important for future depositions is the observation that the same applied potential can result in different currents depending on the elapsed time of the deposition and parameters that are difficult to control precisely. For example, laser-based nozzle pulling systems produce orifices with a small variation in size, and the distance between nozzle and substrate varies when out-of-plane structures are deposited. Therefore, the second part of this article investigates nucleation and growth dynamics under constant currents. To stabilize the current, the applied potential *V* is varied in such a way that the deposition current stays within ±5 % of the set value.

**Figure 3:**
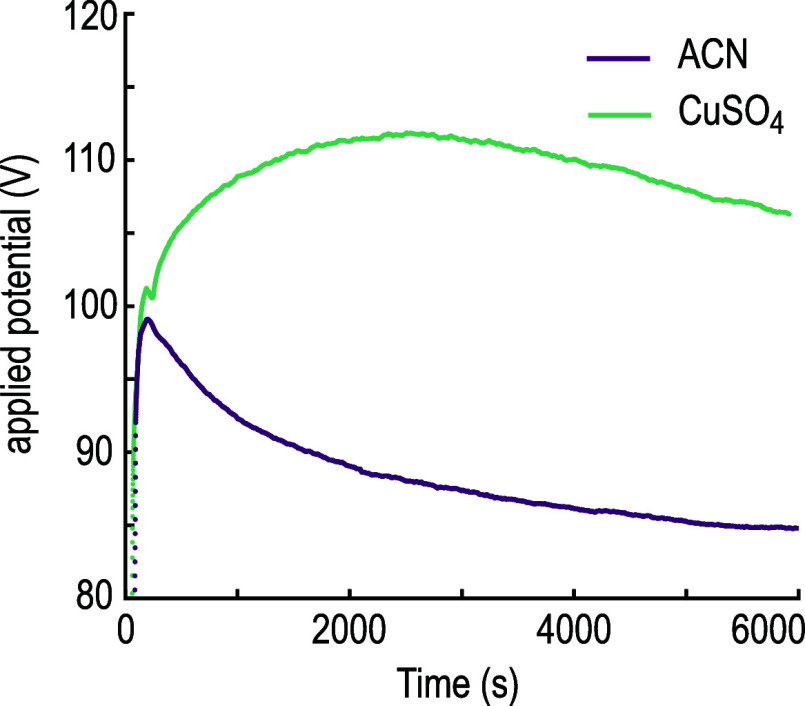
Necessary voltage to stabilize a deposition current of 0.5 nA. To deposit with a constant current, the applied potential has to be adjusted constantly. Here is the necessary voltage shown to achieve a deposition with a constant current of 0.5 nA for ACN (purple) and 1 mM CuSO_4_ in H_2_O (turquoise). Initially, an applied potential of around 100 V leads to a current of 0.5 nA for both solvents. For ACN this potential then starts to drop to 90–85 V. For CuSO_4_, the necessary voltage increases to 110 V and then reduces again. The observed drop in the potential for ACN might be related to an activation of the surface of the Cu electrode (e.g., the dissolution of a passivating oxide layer). The precise reason for the increase for CuSO_4_ remains unclear but could similarly be related to the oxygen evolution reaction on the surface of the Au electrode.

Figure 3 shows the variation in the applied potential to keep a deposition current of 0.5 nA constant for 6000 s. Both acetontrile and CuSO_4_ in water start to deposit at around 100 V, but they require different potentials to keep the current constant. For ACN, the necessary potential drops to 90–85 V, while for CuSO_4_ in water, a higher voltage of up to 110 V is necessary but then decreases again after ca. 2000 s.

### Nucleation and Growth of Cu on Au

**Figure 4:**
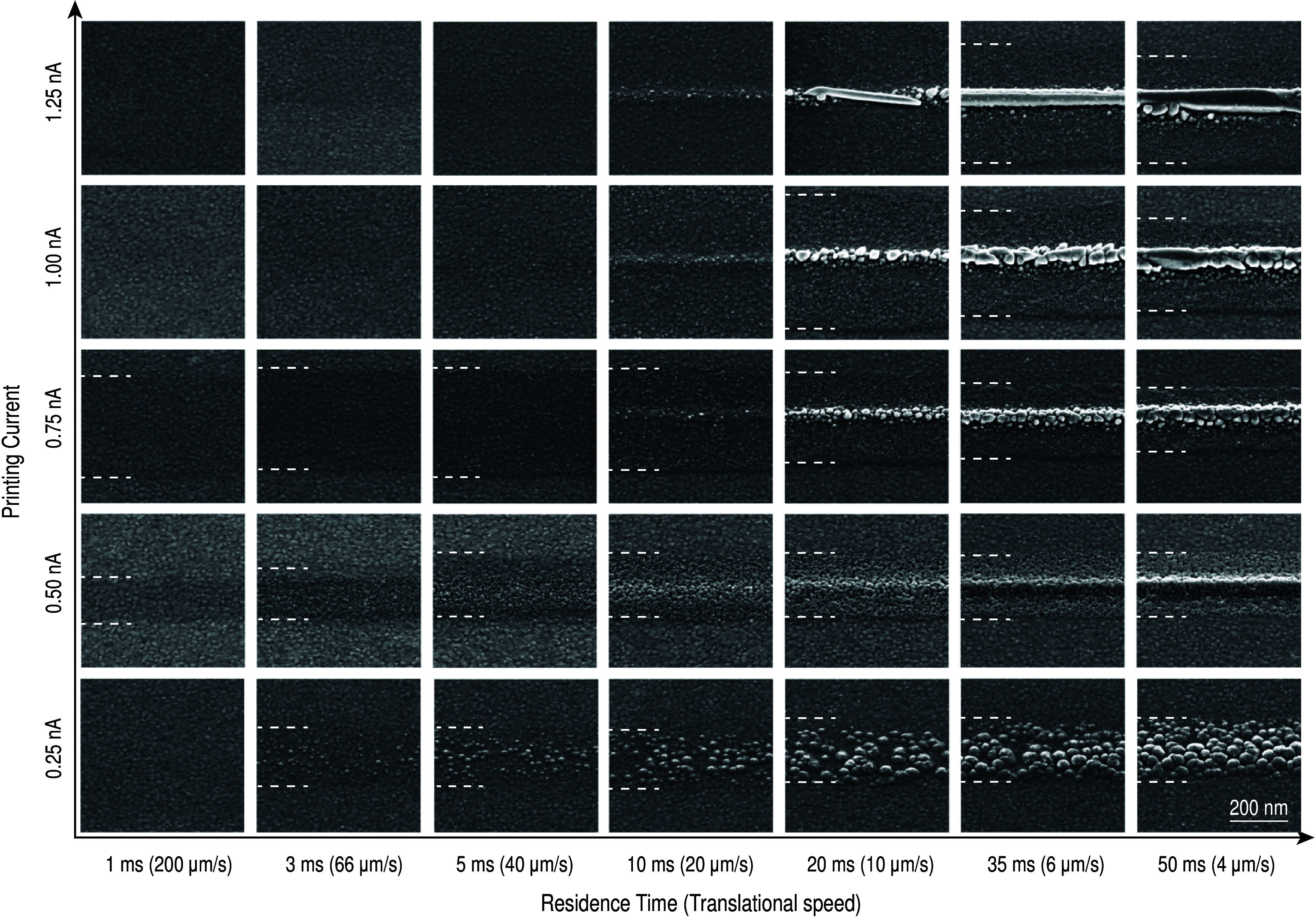
Nucleation of Cu on Au, deposited in ACN. To arrest the deposition at short time steps, lines were printed with different speeds (nozzle-to-substrate distance of 10 μm). We approximate the time stamps for the deposition by using the residence time, which is derived by dividing the orifice size of the nozzle by the line speed. The dashed white lines indicate the area, in which nuclei can be spotted. For 1 and 1.25 nA and residence times <20 ms, this area is larger than the area shown in the micrographs, while for 0.25 nA and 1 ms residence time probably not enough material was deposited to be visible. The substrate surface consists of polycrystalline Au which is visible in the area outside the dashed white lines. A current of 0.25 nA leads to patchy, nonconnected deposits of copper. These patches then increase in size with longer residence time, but the area between the dashed lines remains constant over longer residence times. At 0.5 nA, finer grains are deposited that form a continuous but rough structure at longer residence time. The area in which nuclei can be found also stays constant. For 0.75 nA and higher currents, individual Cu islands can be seen. Progressively fewer but larger grains are observed with increasing current until at 1.25 nA only a single large grain is observed. For residence times below 10 ms, almost no nuclei can be observed for these currents. Also, the area, in which nuclei can be seen, decreases with increasing residence time (from left to right) for currents of 0.75 nA and higher. This reduction in area indicates more localization of the deposition with longer residence times.

**Figure 5:**
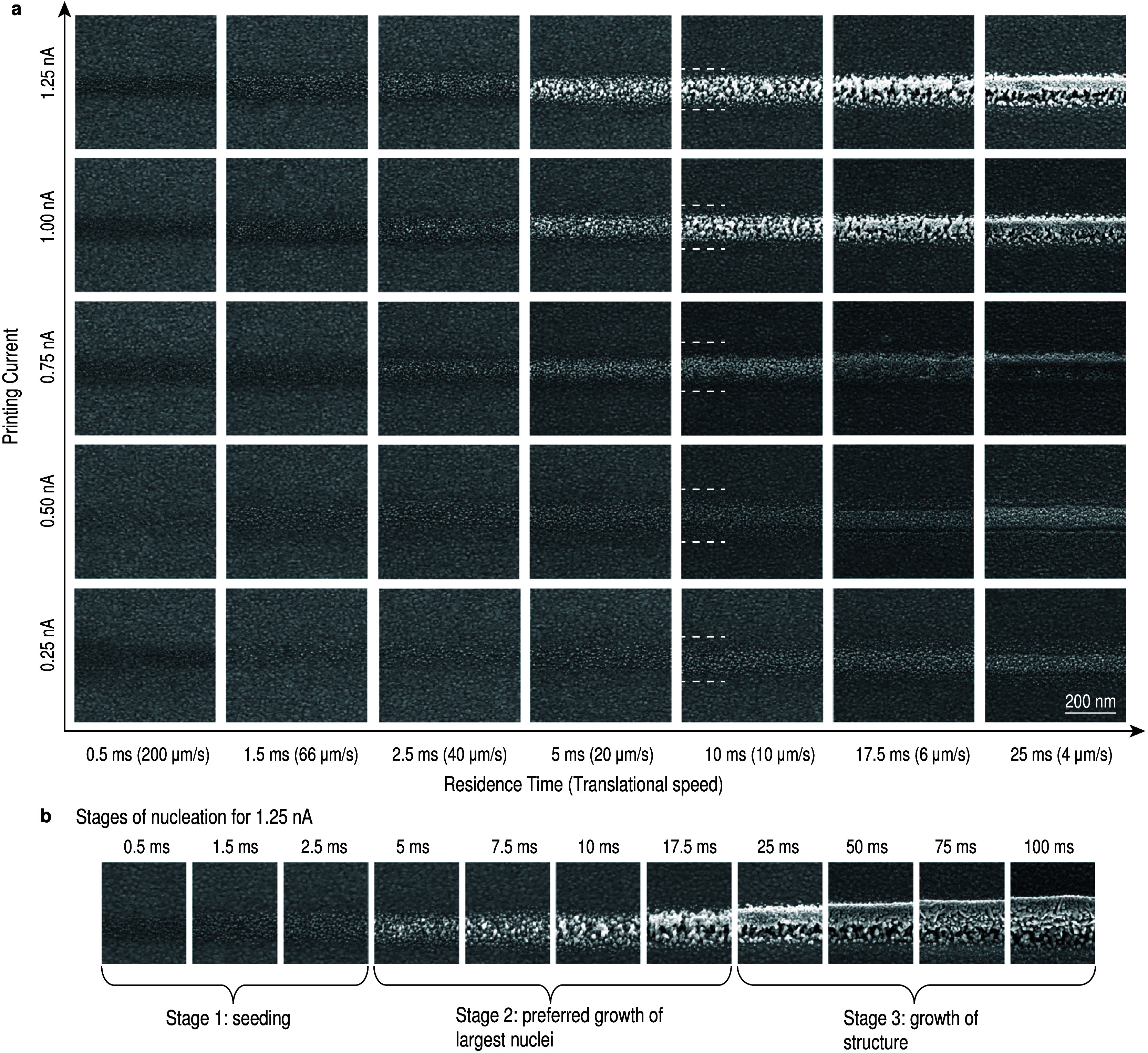
Nucleation of Cu on Au, deposited from 1 mM CuSO_4_in H_2_O. (a) Similarly to ACN, Cu lines were printed using a 1 mM CuSO_4_ solution (nozzle-to-substrate distance of 7.5 μm). The nucleation is shown at shorter residence times (due to the narrower orifice sizes of the nozzles but the same utilized velocity). The dashed white lines again indicate the area in which nucleation is observed. For 0.25 and 0.5 nA, only small grains are observed but no patches as in ACN. At 0.75 nA, the grains coalesce into a continuous but flat structure. For 1 and 1.25 nA, lines consisting of fine grains are observed and not the large crystals that are observed in ACN. At 1.25 nA and 25 ms residence time, a horizontal pore is observed at the interface between substrate and printed Cu line. In general, it appears that less material is deposited (this will be shown in more detail in Figure 7). Furthermore, the area in which nuclei are observed stays constant over different currents and residence times. (b) On the basis of the observed nucleation behavior, we can distinguish different stages of the nucleation for 1.25 nA: First, with short residence times, a number of similar sized nuclei are formed (stage 1). Then, from 5 ms residence time onward, some nuclei grow preferably. This leads to many small and few large grains after 10 ms (stage 2). After around 20 ms the growth behavior changes again: here, the large nuclei start to coalesce (stage 3). This forms a capping layer on top of the porous first layer. This capping layer then grows further with longer residence times.

Figures 4 and 5 show the nucleation of copper on gold substrates printed using ACN and 1 mM CuSO_4_ dissolved in H_2_O, respectively, as a solvent and electrolyte. The nucleation is shown for five different currents (0.25, 0.5, 0.75, 1, and 1.25 nA) and arrested at different time steps. These currents were selected to represent both sections of the CV graph that exhibit different conductances. From previous studies it is also known that droplet-confined electroplating achieves fast deposition rates of around 3 μm s^–1^.^6^ Therefore, the necessary time steps to illustrate nucleation are on the order of a few milliseconds. We found that the most reliable approach to such short time steps is to use a lateral translation of the substrate with different velocities (note that we keep the nozzle stationary but move the substrate). The nominal residence time is then approximated by dividing the orifice diameter (ACN: 200–230 nm; H_2_O: 60–90 nm) by the velocity of the substrate (4–200 μm s^–1^). This approach was chosen as the reaction time of the control software and power source, as well as parasitic capacities induced when switching the system on/off, did not allow us to reliably access such short time steps.

The nucleation behavior of copper in ACN (Figure 4) shows distinct differences between low currents (0.2–0.5 nA) and high currents (≥0.75 nA). The current and the necessary applied potential during deposition are shown in Figure S2. At 0.25 nA, only patches of Cu are deposited. Number and size of the deposited patches increase with increasing residence time. At 0.5 nA, very small grains are observed that successively start to merge with longer residence times and form a rough but continuous structure, as seen from SEM images and later cross-sections. At currents of 0.75 nA and higher, progressively large grains can be observed when the residence time is larger than 20 ms. At 1 nA, few but very large grains can be observed, while at 1.25 nA, dense lines without apparent grain boundaries can be observed. The area over which nucleation is observed (indicated by the dashed white lines when nuclei could be identified) depends strongly on the set current. At low currents, the width of the band stays constant independent of the residence time. At high currents, the nuclei are spread over a larger area (larger than the SE micrograph shows), and the area decreases with longer residence time.

For copper that is deposited using water (Figure 5), generally smaller grains are observed than in ACN. Further, the width of the band in which nuclei are observed stays constant over different currents. For high currents (*I* ≥ 1 nA) after a seeding stage, a few grains grow preferably before merging and forming a layer. This is shown with a higher temporal resolution in Figure 5b. We identify three subsequent stages of the nucleation of Cu in water with 1.25 nA: first, very small nuclei form homogeneously. In a second stage, the largest nuclei grow fastest, until in a third stage, where these grains start to coalesce and form a continuous line. Yet, a porous interface between Au substrate and the Cu line remains.

SI Figure S3 shows the nucleation after the same number of time steps achieved by single or by multiple overpasses of a fraction of the residence time each, using 1 mM CuSO_4_ as the electrolyte. The structures look similar after 10 ms residence time. The results indicate that for 1 nA, using multiple but shorter overpasses leads to an increase in the number of nuclei. Nevertheless, we conclude that using multiple short overpasses does not fundamentally change the growth behavior and the mechanisms during the deposition.

### Illustrating the Voxel during Initial Stages of the Deposition

**Figure 6:**
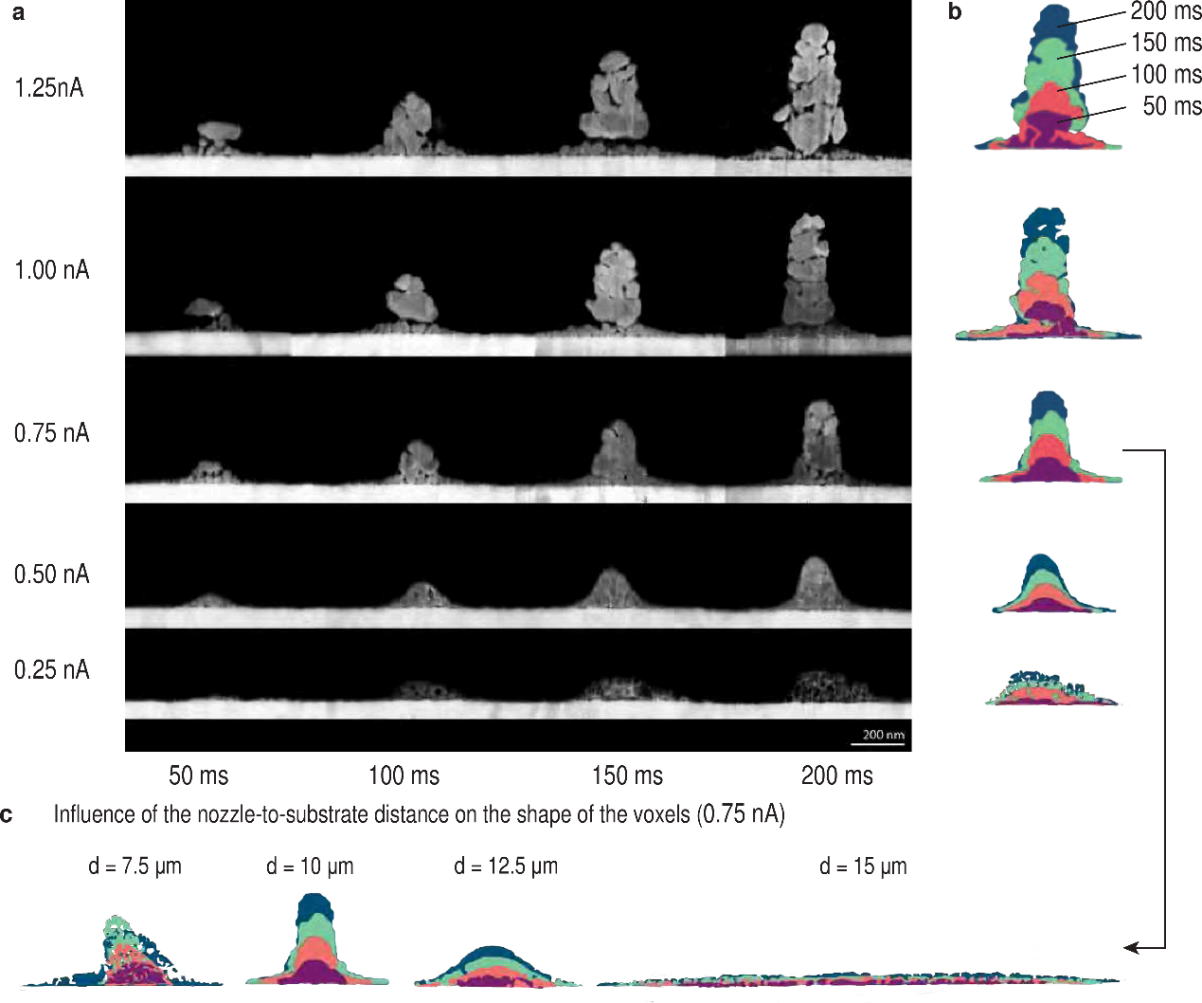
Growth of copper deposited in ACN. (a) Cross-sectional micrographs of Cu lines printed in ACN with currents between 0.25–1.25 nA (nozzle-to-substrate distance of 10 μm). The lines were printed with one or multiple overpasses of 50 ms each (so 150 ms refers to 3 overpasses), cut using a focused ion beam, and imaged in SEM. Cu printed with 0.25 nA exhibits a very porous, low contrast structure resembling a network. With 0.5 nA, a dense structure with a fine-grained size is deposited. With higher currents, structures exhibiting larger grains are deposited. This evolution of the grain structure is in line with the observations from Figure 4 and previous work (in a potential controlled mode).^6^ A current above 0.75 nA increases the lateral thickness of the printed lines, which indicates that higher currents not only deposit more material but also show more porosity between the large grains. Furthermore, currents of 1 nA and higher exhibit a weak connection between substrate and printed structure. The contrast has been modified for the sake of clarity. (b) Overlay of the outlines of different overpasses. The outline of each line was colored and is overlaid to illustrate the evolution of the structure over time. The difference between two overpasses therefore corresponds to the voxel that is deposited within 50 ms. With currents of 0.25 and 0.5 nA, the structure grows over the entire width. For currents above 0.75 nA, the growth is confined to the highest section of the structure. (c) The shape of the voxel, however, depends not only on the current but also on the distance between nozzle and substrate. Here, a series of overlaid outlines is shown for lines that were printed with 0.75 nA and distances between 7.5 and 15 μm. At a distance of 7.5 μm again, a porous structure was observed, probably due to dentritic growth inside a stationary ACN droplet. At a distance of 12.5 μm, the voxels start to become flattened out, and at 15 μm, Cu is deposited onto a large area, probably due to a breakup of the ejected droplet. This leads to a spray of Cu ions instead of confined electroplating. Therefore, the deposition with minimal feature size (i.e. minimal thickness of the structure) requires an optimum between current and distance.

**Figure 7:**
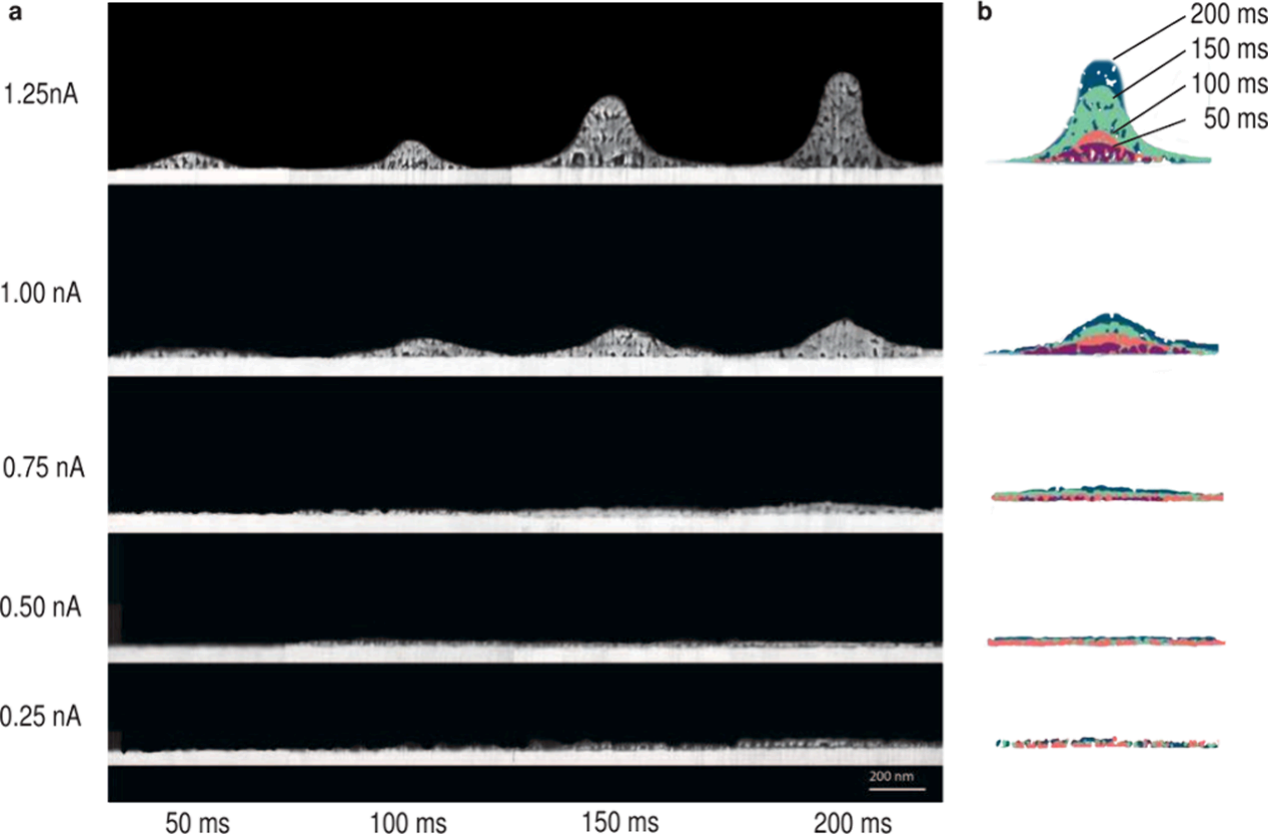
Growth of copper deposited from 1 mM CuSO_4_ in H_2_O. (a) Cross-sections of Cu lines printed with currents between 0.25 and 1.25 nA (nozzle-to-substrate distance of 7.5 μm). The lines were printed with multiple overpasses of 50 ms each (for example, 150 ms refers to 3 overpasses). In contrast to ACN, flat and unconfined deposition is observed for currents ≤0.75 nA. On the basis of the observation in Figure 6c, this indicates that the nozzle was too far from the substrate and the droplets evaporated in-flight.^23^ This leads to a spray and, therefore, unconfined deposition. Currents ≥1 nA show a very fine microstructure with a high porosity. This observed porosity could originate from the formation of H_2_ (which is also indicated by a lower-than-expected volume increase in Figure 8b). (b) Overlay of the outlines of the deposition shows each additional voxel that was printed in 50 ms. Even for high currents, the deposition is not confined to the tip of the structure as for ACN, but growth over the entire width of the structure is observed.

The nucleation of Cu in different solvents and at various currents indicated strong differences in the resulting microstructure. These differences in microstructure are visible in detail in Figures 6a and 7a, that present the cross-sections of Cu lines printed with residence times of 50, 100, 150, and 200 ms at the indicated currents for both solvents. A 50 ms residence time refers to a translational speed of 4 and 2 μm/s with a nozzle with 200 and 100 nm orifice size, respectively. At slower translational speeds, out-of-plane growth of tilted nanowires is observed,^25^ because the out-of-plane growth rate exceeds the velocity of the substrate. Therefore, multiple overpasses of 50 ms each are used to further increase the residence time without introducing out-of-plane growth. Further, this approach allows one to illustrate the material deposited in 50 ms increments.

A current of 0.25 nA and ACN as the solvent deposit, as already shown in Figure 4, only a small amount of material. This carbon-rich structure has a low contrast in SEM. However, it is still visible that this deposit shows a high porosity, resembling a network structure. At 0.50 nA, a dense microstructure is observed. At 0.75 nA, the deposition is confined to a narrower structure (after the first layer), meaning that material is predominantly added to the top section. Further, larger grains than in lower currents are observed. However, porosity starts also to emerge between the grains. A further increase of the current leads to wider (horizontal width) structures, larger grains, and also more porosity in between the grains. In previous reports, a constriction of the base of printed pillars was reported.^6^ This is visible here as well for currents of 1 nA: It appears that the upper part of the structure is not connected to the substrate. This is probably due to the presence of porosity at the interface with a limited number of connecting grains between the substrate and printed structure.

In water, for currents of 0.75 nA and lower (and a nozzle-to-substrate distance of 7.5 μm), a flat, unconfined deposition is observed (referred to as a spraying mode). Spraying occurs when the droplets reach the coulomb limit due to evaporation of the solvent, leading to a breakup of the droplet and hence a deposition in a larger area. Note that currents of 0.75 nA refer to the second regime in the *I*–*V* curves before the slope changes. For currents of 1 nA and higher, a very fine grain structure is observed. This is in agreement with previous reports of Cu pillars printed with this technique^5^ using 1 mM CuSO_4_. At 1.25 nA, the structure exhibits high porosity, especially close to the interface with the substrate. This porosity was expected from Figure 5, and similar porosity has been reported previously.^2^

The shapes of the voxels, however, are a function of not only the current but also the nozzle-to-substrate distance (Figure 6c). A decrease in the distance leads to more porous structure, while at high distance, spraying instead of confined deposition is observed. Therefore, optimal deposition parameter need to balance current and distance to achieve optimized material quality.

**Figure 8:**
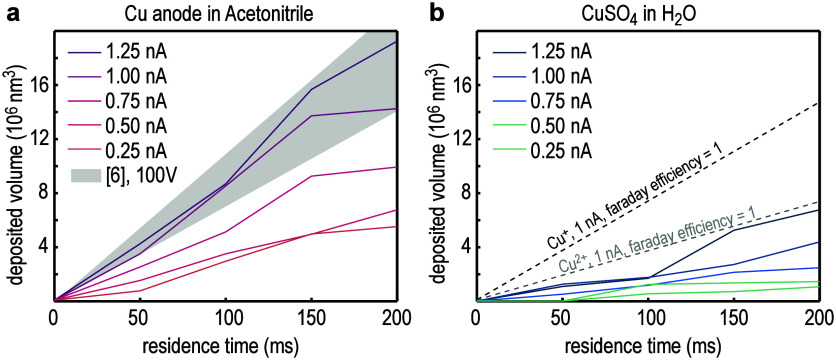
Deposition speed based on cross-sections. (a) The deposited volume was calculated by analyzing the area of the deposit in the cross-sections in Figure 6 and multiplied with 200 nm (based on the definition of the residence time) to obtain the deposited volume for ACN. Here, the deposited volume scales with the current from 4 × 10^6^ nm^3^ in 0.2 s for 0.25 nA (referring to a growth rate of 0.02 μm^3^/s) to 18 × 10^6^ nm^3^ in 0.2 s (growth rate of 0.09 μm^3^/s). Although based on only a single set of crosssections, the calculated deposition rate is in good agreement with previous measurements in a voltage controlled mode^6^ with 100 V. (b) The deposited volume was also calculated for Cu deposited from an aqueous CuSO_4_ solution. Notably, a lower deposition rate was found, ranging from 0.005 μm^3^/s for 0.25 nA to 0.034 μm^3^/s for 1.25 nA.

The observed cross-sections further allow us to calculate the deposited volume. Hereto, the area can be measured and multiplied by 200 nm for ACN and 100 nm for CuSO_4_ (which are the respective nozzle orifice sizes utilized to deposit these structures and are used to calculate the residence time, see SI Table S1 and S2). The porosity was subtracted for the calculation of the area. The measured deposited volumes are shown in Figure 8a,b. For ACN and a current of 0.25 nA, a volume of 4× 10^6^ nm^3^ was deposited in 0.2 s. A high current (1.25 nA) yielded 18× 10^6^ nm^3^ in 0.2 s. This can be translated in growth rates of 0.02 μm^3^/s and 0.09 μm^3^/s, respectively. A comparison with a previous study on the deposition rate of Cu shows a good agreement, where a rate of around 0.1 (0.05–0.11) μm^3^/s was measured when a potential of 100 V was applied. The observed large variation in deposition speed in the previous study might originate from deposition with different elapsed times at the same potential, leading to deposition with varying current and, therefore, large variations in the deposition current. For water, lower deposition rates of 0.005 μm^3^/s (0.25 nA) to 0.034 μm^3^/s (1.25 nA) were determined. Figure 8b also shows the expected deposited volume for a current of 1 nA with a Faraday efficiency of 1 (meaning that all electrons would reduce copper ions) for Cu^+^ (expected in ACN^1^) and Cu^2+^ (expected in water^5^). The observed growth rate of Cu deposited in ACN closely matches the theoretical maximum for Cu^+^. In water, the observed deposition rate is lower than the theoretical maximum for Cu^2+^. This low Faraday efficiency could be caused by side reactions such as the formation of hydrogen from the reduction of H^+^. The formation of hydrogen could also explain the observed porosity in Figure 7.

## Discussion

A possible explanation of the observed current–potential characteristic as well as the differences in nucleation and microstructure of deposited Cu is the transition from a transient to a stationary electrolyte droplet on the substrate.^23^ A transient solvent droplet would evaporate before the next electrolyte droplet arrives. Such a transition has been hypothesized already in previous work based on an observed relationship between applied potential and microstructure^6^ for Cu and ACN but not studied as a function of the current.

At *V* < *V*_ejection_, no droplets are ejected and therefore no current is measured. Strikingly, the necessary potential to start any deposition is up to 2 orders of magnitude higher than in standard electroplating. This is due to the high electric fields necessary to drive the electrohydrodynamic ejection of the droplets. Further, the *V*_ejection_ depends on the orifice size of the nozzle. For optimized deposition performance, an ideal orifice size leads to a linear *I*–*V* curve with a flat slope, which allows fine control of the current and does not lead to a deposition in a spray mode. This ideal orifice size was observed to differentiate among the utilized solvents. The ideal orifice size is probably unique for each solvent system, as electrohydrodynamic ejection depends on a variety of materials parameters of the solvent^4^ (such as the viscosity, surface tension, density, permittivity, and conductivity).

At *V* > *V*_ejection_ and *I* < 0.75–1 nA, droplets are ejected but evaporate quickly, so that the solvent droplet evaporates completely before the next droplet reaches the substrate (or the droplets already evaporate before they impact the substrate^23^), while at (*V* > *V*_ejection_ and *I* > 0.8–1 nA), there is a stationary solvent droplet. For ACN, a current of 1 nA relates to a droplet ejection frequency of 1.73 × 10^7^ Hz, assuming a droplet charge of 360 *e* (droplet radius = 95 nm) as estimated previously.^10^ A stationary solvent droplet could partially screen the electric field between the substrate and the nozzle. This solvent droplet, therefore, acts as an additional resistance in the system, leading to a lower conductance. Further, this hypothesis explains the observed changes in the nucleation between *I* ≤ 0.5 nA and *I* ≥ 0.75 nA. Electroplating in a stationary droplet is less kinetically dominated and therefore leads to thermodynamically more favorable microstructures (i.e. larger grains), as observed for high currents in ACN. In water, however, the trend toward larger grains is less visible. This could be due to higher levels of contamination (leading to increased nucleation rates) or the formation of hydrogen on the substrate.

An alternative explanation for the observed different conductances could be a change in the number of charges per droplet. The ejection of the droplets is driven by the repulsive force between equally charged ions overcoming the surface tension of the solvent at the tip.^1^ This ejection process is physically best described by first analyzing the critical charge of an isolated droplet (coulomb limit). Here, the critical charge is defined as^26^
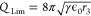
with γ the surface tension of the solvent, ϵ_0_ the permittivity of free space, and *r* the droplet radius, with the last being linked to the orifice size of the nozzle.^23^ A higher charge than *Q*_Lim_ would lead to disintegration of the droplet due to repulsive forces. At the nozzle, the ejection of a droplet usually occurs when the very tip reaches half this critical charge.^10,25,27^ We therefore concluded that an increased applied potential/higher current cannot change the number of charges per droplet, as this number is rather tied to orifice size and surface tension of the solvent. A higher current must therefore increase the frequency of the ejection of droplets rather than their charge. Further, we rejected the explanation of a change in the ejection mode.^4^ There are different EHD modes described in the literature, which are in order with ascending necessary electric field: dripping mode, pulsating cone, cone-jet, tilted-jet, and twin- or multijet. Yet, only the dripping mode was observed in previous studies.^23^

The decrease in necessary potential to drive the ejection over time could originate from the dissolution of a passivating layer on the anode or the generation of a surplus of charge carriers (i.e. more charge carrier generated than ejected, possibly due to the involved high potentials), which then increase the conductivity of the solvent. The second hypothesis is further supported by the observation that the necessary *V*_ejection_ is higher in pure water that in a solution of 1 mM CuSO_4_. Therefore, and unsurprisingly, a higher number of charge carriers increases the observed current at a given potential.

## Conclusions and Outlook

Droplet-confined electroplating must be both predictable and controllable to make it a versatile fabrication technique. Therefore, we investigated the current–potential relationship and explored how the current can serve as an operando feedback mechanism for the deposition. An analysis of the current unraveled a characteristic *I*–*V* curve. This curve allows us to investigate and quantify the influence of a variety of parameters, such as the elapsed time, the distance between nozzle and substrate, and the orifice size on the deposition. The deposition at constant currents allowed the nucleation and growth of copper in both water and ACN as an electrolyte.

For ACN, a current of 0.75 nA indicates the best shape of the voxel and a dense microstructure, although higher currents allow for lines without visible grain boundaries. An analysis of the current indicates that this is a transient regime for both studied electrolytes, where the droplets potentially still evaporate before the next droplet impacts. At a current of 1 nA, the growth inside the solvent droplet leads to large (and sometimes very long) grains. For water, the volumetric deposition speed is much lower at the same current due to copper forming a divalent species in water and possibly due to other charge carriers, such as protons getting reduced on the substrate and forming hydrogen. The volumetric deposition rate as well as the out-of-plane growth rate have only been determined for initial printing stages. Future work is necessary to confirm the result also found for taller and intricate structures. Further, improved procedures to calibrate the distance between nozzle and substrate (or nozzle and printed structure, for taller out-of-plane structures) are necessary, for example based on the formation of a meniscus between nozzle and substrate/structure (similar to scanning electrochemical cell microscopy (SECCM)).^28^

The results presented here could also serve as a template protocol for testing novel candidate solvents for EHD-RP: first, a CV curve is measured to optimize the orifice size for the given solvent, similar to Figure 2f. Then lines are printed and cross-sections are analyzed to determine if material has been deposited. No deposition but a measured ejection current can indicate no dissolution of the sacrificial anode^2^ or a decomposition of the electrolyte. If deposition in a spraying mode is observed as in Figure 7 for low currents, the distance should be reduced, while dendritic growth indicates too close a distance, too high a current (Figure 6), or an insufficient vapor pressure of the solvent that prevents timely evaporation of the solvent. An iterative approach will allow to quickly optimize the deposition parameters (orifice size, set current, and distance between nozzle and substrate) for a chosen solvent and metal combination. Such a study will provide insights into the interplay and importance of dielectric constant, surface tension, and vapor pressure of the candidate solvent for successful droplet-confined electroplating.

In conclusion, the current offers a self-evident tool to predict the properties of deposited copper. Since any slicing algorithm requires knowledge about the geometrical shape and volume of an individual voxel, utilizing the current as an indicator for the volumetric deposition rate is crucial for the controlled fabrication of intricate three-dimensional shapes.^10^ This significantly readies the technique to deposit functional structures.

## Experimental Section

### Materials

Nozzles for deposition were fabricated on a P-2000 micropipet puller system (Sutter Instruments) from filamented quartz capillaries (Sutter Instruments, item QF100-70-15). Nozzle diameters were determined on a Quanta 200F instrument (Thermo Fisher Scientific), equipped with a Schottky-type field emission gun (FEG) in low vacuum mode (30 Pa) to avoid charging. Nozzles with diameters of 60–90 nm were used for salt solutions and 200–230 nm for ACN. The nozzles were filled with ACN (Optima, Fisher Chemical) or copper sulfate (1 mM CuSO_4_ (Sigma-Aldrich, 99.999% metal basis) in water (LC/MS-grade, Fisher Chemical)) by using gas-tight glass syringes with custom made PEEK tips. The nozzles were cleaned inside by rinsing with the solvent. Substrates were 0.4 × 2.0 cm pieces of Au-coated (80 nm) Si wafers. The substrates were cleaned in technical acetone and analytical isopropanol and subsequently blow-dried with compressed air before use. Cu wires (Alfa Aesar, 0.25 mm diameter, 99.999% metal basis) were etched in concentrated nitric acid (65% HNO_3_, Sigma-Aldrich) for 10 s, dipped in water, and stored in analytical ethanol until use (maximal 10 min), while Au wires (Metaux Precieux SA, 99.999%) were immersed for 30 s, dipped in water, and stored in air.

### Setup

Fundamentally, the setup consists of piezo stages that move the substrate in the *X*, *Y*, and *Z* directions (QNPXY-500, QNP50Z-250, Ensemble QL controller, Aerotech). Additionally, stage translations in *X* and *Y* direction larger than 500 μm were enabled by additional long-range stages (M112-1VG, PI for the *Y* direction, manual micrometer screw, Mitutoyo for the *X* direction). Piezo stages and the power source were controlled through a custom Matlab script. The nozzle is mounted on a motorized nozzle holder (Z825B, controlled with Kinesis, both Thorlabs). The deposition is observed through an optical microscope composed of an 50× objective lens (LMPLFLN, Olympus) and a CMOS camera (DCC1545M, Thorlabs). The lens is mounted at an inclination of 60° to the substrate normal, and the substrate is illuminated from the behind using a green LED (LEDMT1E, Thorlabs). The green light (λ = 530 nm) and a NA = 0.5 yields a resolution limit for the optical system of 530 nm. A power source (B2962, Keysight) with triaxial cable connectors was used for polarizing the anodes. The metal wires were connected by using a mechanical clamp. The complete printing setup is mounted inside a gas-tight box to provide an oxygen-free atmosphere (controlled with an oxygen sensor, Module ISM-3, Dansensor) and is placed on a damped SmartTable (Newport) to provide a vibration-free environment.

### Deposition

Deposition was performed at room temperature in an argon atmosphere (<100 ppm of O_2_). Typical potentials applied to the anode during printing were 80–160 V. For printing with a certain current, the potential was adjusted to match this ejection current by averaging the current over 40 data points (spaced 0.2 s) and adjusted in 0.2 V increments if the average deviated more than 5%. Nozzle substrate distance was controlled to be 5–15 μm by analyzing the light microscope images and adjusting the position of the *z*-axis piezo stage accordingly.

### Analysis

High resolution scanning electron microscopy (SEM) was performed with a Magellan 400 SEM (Thermo Fisher Scientific, former FEI) equipped with an Octane Super EDXsystem (EDAX, software: TEAM). Tilt angles were 55°. HR-SEM images were taken in immersion mode with an acceleration voltage of 5 kV. A dual-beam Helios 5UX (Thermo Fisher Scientific) with a focused Ga^+^ liquid metal ion source was used for the focused ion beam (FIB) milling of cross-sections. Prior to FIB milling, the pillar was coated by a protective carbon layer, which is visible in the SEM image as a black background.

## Supporting Information Available

Supporting Information: . (PDF) The Supporting Information is available free of charge at https://pubs.acs.org/doi/10.1021/acselectrochem.4c00085.

Additional current measurements supporting Figures 1 and 2, *I*–*V* curves during nucleation in Figures 4 and 5, additional SEM images detailing the difference in nucleation of Cu deposition with multiple overpasses, and calculation of the deposited volume for Figure 8 (PDF)
