# Supplementary material for: Droplet-Confined Electroplating for Nanoscale Additive Manufacturing: Current Control of the Initial Stages of Growth of Copper Nanowires
Source: ACS Electrochem. 2024 Nov 4;1(2):205–15. doi: 10.1021/acselectrochem.4c00085 (PMC11808642; doi:10.1021/acselectrochem.4c00085)
Supplement: Supplementary file 1 — ec4c00085_si_001.pdf [file ec4c00085_si_001.pdf]

Supplementary Information

Droplet-Confined Electroplating for Nanoscale Additive Manufacturing: Current Control of the Initial Stages of Growth of Copper Nanowires

Mirco Nydegger and Ralph Spolenak*

Laboratory for Nanometallurgy, Department of Materials, ETH Zürich, Vladimir-Prelog-Weg 1-5/10, Zürich 8093, Switzerland

*E-mail: ralph.spolenak@mat.ethz.ch

Table of Content

Calculation of the deposited volume S2
Additional current measurements S3
I-V curves during nucleation S4
Difference in nucleation of Cu deposition with multiple overpasses S4

Calculation of the deposited volume

The areas for the calculation of the deposited volume were measured with ImageJ (after a binarisation) and are given in the following two tables. These values are then divided by 1.0972 (square of 1,0475, the number of pixel per nm) and multiplied with the nozzle diameter (to achieve a consistency with the residence times).

	0.25 nA	0.50 nA	0.75 nA	1.00 nA	1.25 nA
50 ms	3823	8029	13510	18856	22941
100 ms	15847	18866	27755	46432	47257
150 ms	26922	26791	50389	74882	85599
200 ms	29900	36722	54084	77805	105225

Table S1: Deposited volume for acetonitrile. Measured area in pixel after substracting the porosity from the crosssectional images in Fig. 5.

	0.25 nA	0.50 nA	0.75 nA	1.00 nA	1.25 nA
50 ms			5579	13781	11912
100 ms	5953	13573	12885	19322	18584
150 ms	7711	14831	23492	29874	57794
200 ms	11653	15900	27200	48293	74522

Table S2: Deposited volume for copper sulphate. Measured area in pixel after substracting the porosity from the crosssectional images in Fig. 6.

Calculation for the faraday efficiency:

Faraday's law of electrolysis describes the volume V deposited from a charge q as

$$V = \frac{M}{\rho}(\frac{q}{z*F})$$

with M the molar mass in kg/mol, ρ the density in kg/m^3,q the Charges ($1\ nA * 0.2s = 2 * 10^{-10}$) in C, F the Faraday's constant in C/mol, and z the valency number of the cation to be deposited, which is either 1 for Cu^+ or 2 for Cu^{2+}. Therefore, the Volume deposited in 0.2 s is given by the following equation:

$$V = \frac{63.546*10^-3}{8.96*10^3}(\frac{2*10^{-10}}{z*96485})$$

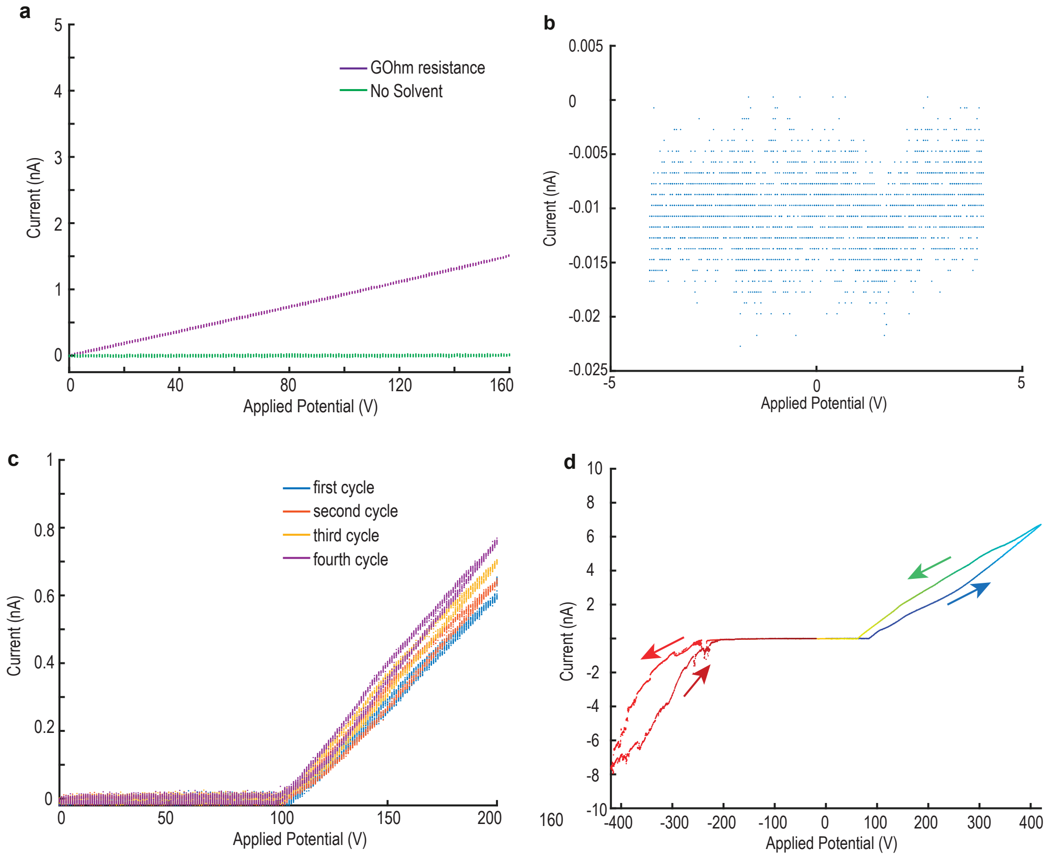

Figure S1: Additional current measurements. Here, an additional set of current measurements is given to support the statements made in the article. **(a)** First, it is ruled out that the setup causes the observed potential-current behaviour. For example, a change in the internal resistance in the ampere-meter could lead to a similar change in conductance. Yet, when connecting a 100 GOhm resistor instead of the nozzle no change in the conductance is observed. Further, no parasitic currents could be observed when the potential is swept on a nozzle without solvent. **(b)** Potential-sweep around 0 did not indicate any current that could originate from a dissolution of the Cu anode in ACN (Cu Anode, ACN, 10 µm distance, 50 mV/s). Therefore, without ejection (similar to an open-circuit state) no significant dissolution takes place. **(c)** Cycling multiple times between 0 and 160 V leads to an increase in the current for a given applied potential (above the minimal ejection potential). This could be driven by formation of surplus charge carrier in previous cycles or an activation of the surface, e.g. by a dissolution of a passivation oxide layer. **(d)** Full range potential sweep: The potential range of the utilized power supply is limited to $\pm$ 420 V. We swept this range from 0 to 420 to -420 to 0 Volts with 500 mV/s. Interestingly, the descending node of the current (after reaching 420 V, reducing towards -420 V) indicated a higher current than in the ascending node (from 0 to 420 V increasing). Again, this could be due to the generation of surplus charge carriers or an activation of the surface. Ejection with a negative potential (effectively inverting anode and cathode) is possible, yet it requires a higher minimal potential of -200 V to start the ejection. Further, the ejection is unstable and the current shows large variations. The origin of the variations is not known. So far, no successful depositions have been carried out with EHD-RP utilising a negative applied potential.

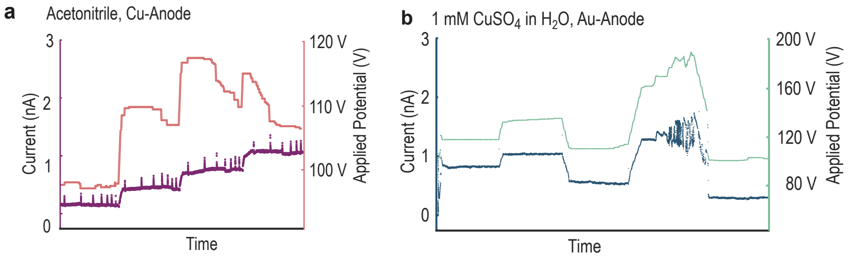

Figure S2: I-V curves during nucleation. (a) The current (left axis, purple) and potential (right axis, orange) curve during deposition of lines used to image the nucleation in acetonitrile with a sacrificial Cu anode (the curve for 1.25 nA is missing). The regular peaks of the current are induced by closing relays (connecting the anode to the power supply), as the wires were electrically isolated during regular distance calibrations. These calibrations ensured a correct distance between nozzle and substrate during the deposition. Interestingly, the necessary potential decreased during the deposition to ensure a constant current. This indicates an reduction of the resistance of the system. This could probably be caused by an easier dissolution of the sacrificial anode. **(b)** The current (left axis, blue) and potential (right axis, turquoise) curve during the deposition of the lines used to image the nucleation for 1 mM $CuSO_4$ in H_2O. In contrast to ACN, no reduction in the necessary potential to keep the current constant was observed. For deposition with an planned current of 1.25 nA, a large increase in the potential is observed. Further, the current is unstable and exhibits large variations.

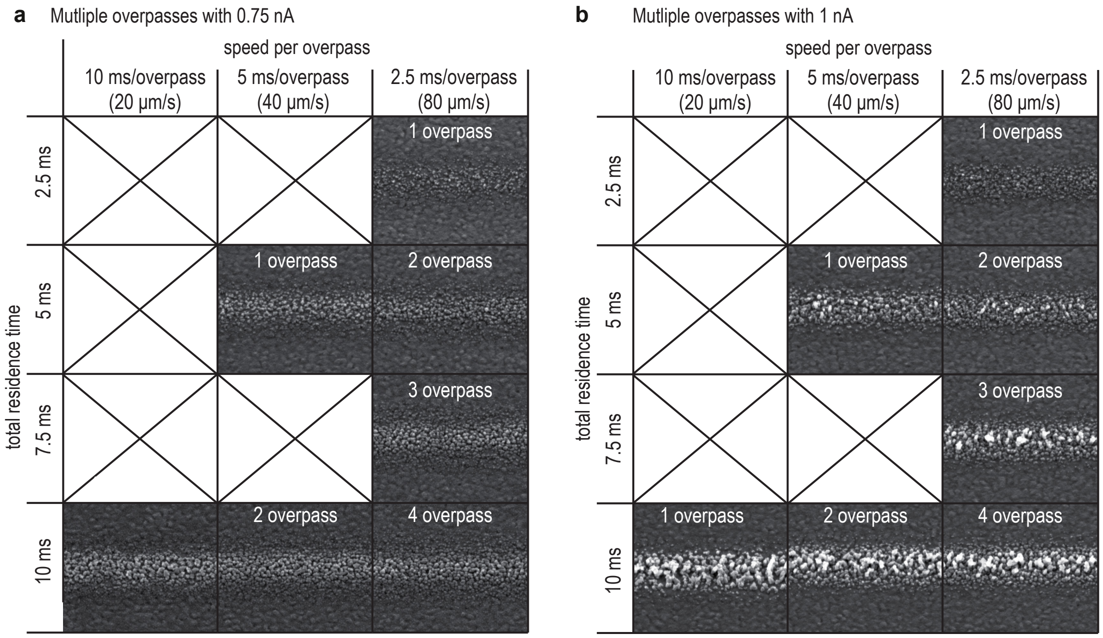

Figure S3: Difference in nucleation of Cu deposition from 1 mM $CuSO_4$ between single vs multiple overpasses with equal total residence time. The nucleation is shown for a current of 0.75 nA in **(a)** and for 1 nA in **(b)**. The residence time is derived by dividing the orifice size of the nozzle by the velocity of the substrate during a translational movement. A total residence time of 10 ms is compared for 1 overpass, 2 (5 ms each) and 4 (2.5 ms each). for both currents no fundamental differences can be seen. It seems, however, that more and finer grains are observed for multiple overpasses. This could originate from new nuclei being formed in a second overpass and less time to form more stable larger grains.
